# Using median survival in meta-analysis of experimental time-to-event data

**DOI:** 10.1186/s13643-021-01824-0

**Published:** 2021-11-02

**Authors:** Theodore C. Hirst, Emily S. Sena, Malcolm R. Macleod

**Affiliations:** 1 Centre for Clinical Brain Sciences, University of Edinburgh, Edinburgh, UK; 2 Department of Neurosurgery, Royal Victoria Hospital, Belfast, UK

**Keywords:** Systematic review, Meta-analysis, Median survival, Time-to-event, Experimental studies

## Abstract

**Background:**

Time-to-event data is frequently reported in both clinical and preclinical research spheres. Systematic review and meta-analysis is a tool that can help to identify pitfalls in preclinical research conduct and reporting that can help to improve translational efficacy. However, pooling of studies using hazard ratios (*HR*s) is cumbersome especially in preclinical meta-analyses including large numbers of small studies. Median survival is a much simpler metric although because of some limitations, which may not apply to preclinical data, it is generally not used in survival meta-analysis. We aimed to appraise its performance when compared with hazard ratio-based meta-analysis when pooling large numbers of small, imprecise studies.

**Methods:**

We simulated a survival dataset with features representative of a typical preclinical survival meta-analysis, including with influence of a treatment and a number of covariates. We calculated individual patient data-based hazard ratios and median survival ratios (*MSR*s), comparing the summary statistics directly and their performance at random-effects meta-analysis. Finally, we compared their sensitivity to detect associations between treatment and influential covariates at meta-regression.

**Results:**

There was an imperfect correlation between *MSR* and *HR*, although the opposing direction of treatment effects between summary statistics appeared not to be a major issue. Precision was more conservative for *HR* than *MSR*, meaning that estimates of heterogeneity were lower. There was a slight sensitivity advantage for *MSR* at meta-analysis and meta-regression, although power was low in all circumstances.

**Conclusions:**

We believe we have validated *MSR* as a summary statistic for use in a meta-analysis of small, imprecise experimental survival studies—helping to increase confidence and efficiency in future reviews in this area. While assessment of study precision and therefore weighting is less reliable, *MSR* appears to perform favourably during meta-analysis. Sensitivity of meta-regression was low for this set of parameters, so pooling of treatments to increase sample size may be required to ensure confidence in preclinical survival meta-regressions.

**Supplementary Information:**

The online version contains supplementary material available at 10.1186/s13643-021-01824-0.

## Introduction

Many types of biomedical research use time-to-event data—this is particularly prominent in cancer research which rightly attracts considerable focus given the significant economic and health burden that it inflicts on society throughout the world [1, 2].

Meta-analysis is a tool to combine data from multiple studies in order to increase the precision of an estimation of treatment effect. It is used commonly in clinical practice to combine data from large clinical trials and is as such heralded among the highest levels of evidence in medicine [3, 4]. It also allows for the assessment of heterogeneity—that is, investigation of the source of variation of estimates given in individual studies. Preclinical studies are a pillar in the ‘bench to bedside’ process and are vital in informing the development of novel treatments throughout medicine.

However, translation of treatments from the preclinical to the clinical phase is known to be inefficient [5, 6]. Systematic review and meta-analysis is a tool to thoroughly summarise the literature pertaining to a particular area or treatment and identify pitfalls that might explain translational efficiency: typically, these include limitations in external or internal validity, and publication bias [7–9]. Investigation or explanation of heterogeneity is the key step in a meta-analysis of preclinical studies. This is frequently done using meta-regression, although this is likely limited by low sensitivity and therefore type II errors are an issue in result interpretation [10, 11]. Furthermore, collinearity is often present in preclinical survival datasets, so multivariate meta-regression has a theoretical advantage in identifying and accounting for interactions between predictors. However, this is thought to come at the cost of further compromise in sensitivity. Publication bias is an issue almost universally encountered in preclinical meta-analyses—while tools to estimate its effect have been developed, they may underestimate its influence [12, 13].

Analysis of time-to-event data typically focuses on the generation and comparison of hazard functions—most commonly via log-rank or Cox proportional hazards methods [14]. The hazard function is the most popular assessment method for survival data because it is versatile, allows for the inclusion of censored data (i.e. individuals for whom the event of interest does not happen during the study or those who drop out of the study early) and provides a single metric that represents the risk of event occurrence throughout the observation period [15, 16]. Similarly, hazard ratios (*HR*s) are deemed the gold standard summary statistic for use in the meta-analysis of clinical trials [17]. However, the calculation and pooling of precise *HR*s for meta-analysis is only possible by either obtaining individual patient data (IPD) or if measures relating to hazard function are reported directly in each included study. The former is impractical because of the time and resources required in obtaining and handling individual patient data, and the second is unfeasible because relevant hazard data is only presented in a minority of clinical trial manuscripts [18].

In preclinical studies, there is no established gold standard summary statistic: calculation of hazard ratios is particularly challenging and at present there is no statistically validated alternative. The relevant information for use of *HR*s is rarely included in manuscripts and contacting study authors for induvial animal information cannot be relied on as response rates to direct communication can be inconsistent. Furthermore, preclinical meta-analyses typically consist of a large number of small studies which would compound the issues with IPD meta-analysis discussed above. While there are methods reported to estimate hazard functions from Kaplan-Meier graphs [19], this process is cumbersome when compared with the collection of other outcome data, such as those relating to volume or functional performance scales. Other methods of *HR* estimation are reported but are generally laborious or require specific programming [20–22].

There are other metrics that can be used to summarise survival data. The simplest, and most intuitive, is the median survival; this is frequently if not universally used when describing survival datasets, for example for clinical trials. It is much simpler to generate accurately than the other methods and can easily be measured from a Kaplan-Meier chart of any study size. The significant limitations, however, are the lack of an inherent measure of spread and a concern that median survival may not accurately represent the entire observation period—for example, a difference in long-term survivorship may not be accounted using this metric but would be detected with hazard-based analyses. Odds ratio or risk ratio-based summary statistics have the same problem [20].

Michiels et al. [23] compared meta-analyses of clinical trial data using IPD-derived *HR*, an odds ratio-based approach (comparable to the Kaplan-Meier *HR* estimation tool described in [19]), and median survival ratio (*MSR*) as a summary statistic. They found comparable global estimates of effect for each method, and on this basis, we developed a technique for pooling survival data using median survival ratio, with the number of animals per experiment used for weighting in place of inverse variance [24]. It appears to have given sensical results in preclinical meta-analyses [25–27], especially when compared to prior metrics [28]. Michiels did, however, observe a proportion of *MSR*s favouring treatment/control oppositely to their counterpart *HR*s, and on this basis, the authors advised against the use of *MSR* in clinical meta-analysis.

It could be argued that these findings are not relevant to animal data—the authors pooled data from a small number of medium-sized clinical trials (*n* in the hundreds), where a single *MSR* figure may be more, or less, representative of the dataset as a whole than for small animal studies. The weightings between studies were relatively constant and study sizes ranged much less than for animal experiments encountered in prior glioma meta-analyses (*n =* 3–30 per group). Heterogeneity in preclinical meta-analyses is generally much higher than that seen in this meta-analysis. Crucially, the authors did not compare performance on the investigation of heterogeneity. Thus, there is limited applicability of their conclusions to preclinical meta-analysis, where the focus falls on the investigation of heterogeneity rather than the magnitude and precision of efficacy estimates.

In this study, we therefore aimed to assess *MSR* as a tangible, practicable summary statistic when compared with IPD-derived *HR*. Secondly, we aimed to compare the performance of each summary statistic at meta-analysis in terms of the detection of overall treatment effects, between-study heterogeneity and effects of covariates at both univariate and multivariate meta-regression. Finally, we aimed to assess the impact of a publication bias effect on these simulations. We chose to undertake a simulation because of the significant noise, heterogeneity and difficulty of *HR* calculation in real animal datasets. Furthermore, simulation studies allow for the control of the underlying data and a prior knowledge of key features—thereby allowing for a more rigorous methodological assessment [29, 30].

## Methods

### Survival simulation

All simulations were undertaken in Stata 16 (Statacorp, USA) on a Microsoft Surface Pro 7 laptop. Code is provided in Supplementary Material 1.

We first planned to simulate a single large meta-analysis consisting of 1000 experiments. Experience of previous survival meta-analyses suggested a median *n* = 8 per group, with a range around 3–30 [25]. We therefore created 16,000 individuals, assigning each randomly to one of 1000 studies. This was done using a beta function to give a positively skewed distribution of study sample sizes (as seen in prior survival meta-analyses). Individuals were divided into control and treatment groups alternately within each experiment to give roughly equal group sizes.

Following this, we created 10 more variables—to represent typical study design and quality features assessed in preclinical meta-analyses. We created 5 categorical variables (each consisting of 5 options), 3 binary variables and 2 continuous variables. We generated random values (uniformly distributed between 1 and 5 for categorical variables and 0 and 1 for binary variables, and with a normal distribution for continuous variables) and assigned these to each experiment group.

We then used the *survsim* function in Stata to generate survival data for each of the 16,000 individuals, relating to both event occurrence and time to event [29]. In this, we incorporated predictive values of treatment and associated influence of 7 predictors on treatment outcome (4 categorical, 2 binary, 1 continuous) into the model. The remaining 3 predictors were kept as controls—that is, with no influence on survival. The survival times were scaled to an arbitrary timeframe of 50 days at which the majority of individuals would have died. We used Cox regression to confirm the influence of treatment and of each variable on survivorship.

For each of the 1000 experiments, we used the *stcox* function to calculate log-*HR* (ln*HR*) and associated SE for each experiment, using a maximum likelihood, proportional hazards-based iterative approach. Similarly, log-*MSR*s (ln*MSR*) were calculated for each experiment. Because this does not provide any inherent measure of variance, we estimated SE using 1/√*n* [24].

We pooled experiments using DerSimonian and Laird Random Effects meta-analysis [31], via the *metareg* command. We chose a random-effects model because of the innate heterogeneity of study design and conduct seen in the animal literature, as well as the large *I*^2^ values typically encountered in preclinical meta-analyses. Using the *τ*^2^ estimate, attained via a method of residual maximum likelihood, we were able to calculate both fixed and random-effects weighting for each summary statistic for each experiment.

### Meta-analysis and meta-regression sensitivity assessment

Next, we looked to examine the performance of each summary statistic on application to meta-analysis. Points of interest were the sensitivity of each measure to detect a global efficacy estimate, between-study heterogeneity (as defined by *τ*^2^ and *I*^2^ measures), and most importantly for the detection of associations between treatment effect and each of the predictive variables at both univariate and multivariate meta-regression. Furthermore, we looked to appraise the sensitivity across a range of meta-analysis sizes comparable to those encountered in preclinical meta-analyses.

In order to achieve this, we extended the first dataset. Using the same parameters as above, we simulated 100,000 experiments including 1.6m individuals. Each experiment was randomly assigned to meta-analysis groups of sizes 20, 50, 100, 200, 500 and 1000 studies. *HR*- and *MSR*-based summary statistics were calculated as described above.

We then performed a meta-analysis of each group (including between 20 and 1000 studies), applying a limit of 1000 such that there were 1000 meta-analyses of sizes 20–100 experiments, 500 of size 200, 200 of size 500 and 100 of size 1000. Ideally, we would have undertaken 1000 of each group although this was impractical due to computing limitations. After this, we counted the number of meta-analyses returning significant (*α* = 0.05) treatment effect for each (a global efficacy estimate greater than 0), as well as mean ± *SD* of *I*^2^ and efficacy estimate for each meta-analysis size using both summary statistics.

Finally, we compared the sensitivity of meta-regression to detect associations between predictive variables and treatment outcome. This was done for the full range of meta-analysis sizes and for both summary statistics, using both univariate (*α* = 0.005 after Bonferroni correction) and multivariate (*α* = 0.05) strategies.

### Publication bias assessment

Using the large dataset, we undertook a log-rank test (*α* = 0.05) to determine whether a significant treatment effect would be observed in each experiment. Following this, we created daughter datasets in which a set proportion (0%, 25%, 50%, 75% and 100%) of the non-significant studies were selected at random and discarded, thereby creating a series of large datasets with every parameter identical other than the influence of a file drawer effect.

Following this, we proceeded with meta-analysis in the same way as above but using only *MSR* data. At the meta-regression stage, we chose to compare the performance of multivariate meta-regression only because of the findings in earlier stages of the study.

## Results

### Survival simulation

Our simulation returned 1000 studies with median 8 animals per group, *IQR* 7–10, range 2–23 (see Supplementary Material 2). During the simulated follow-up period (arbitrarily scaled to 50 days), 15,804 (98.8%) deaths occurred, with 196 (1.2%) surviving the duration of the experiment. The overall median survival was 8.58 days.

**Fig. 1:**
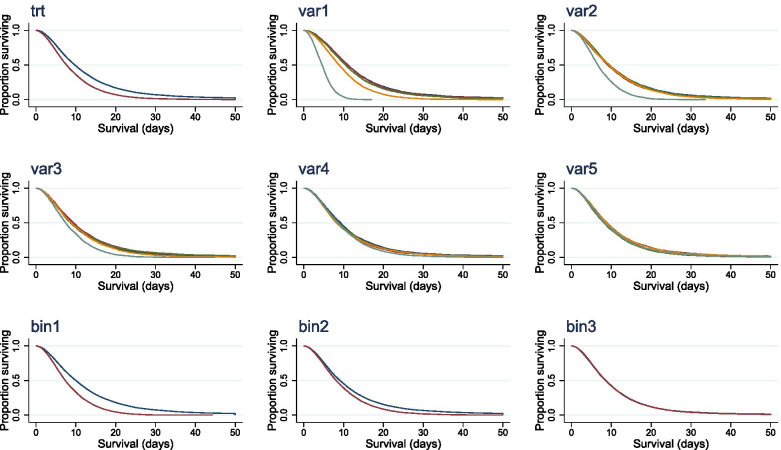
Survival curves for simulated data. Kaplan-Meier plots for simulated individuals categorised by treatment group (trt), categorical variables (Var1-5) and binary variables (Bin1-2)

Cox regression of the individual data (via *stcox* command) suggested a significant treatment effect (*HR* 1.50 ± 0.024, *Z* = 24.8, *p* < 0.001), with a corresponding median survival ratio (*MSR*) of 1.27 (Fig. 1). Furthermore, the influence of all 7 variables intended to impact on survival was demonstrable on a multivariate Cox regression. Kaplan-Meier curves visualising survival stratified by each categorical variable group are shown in Fig. 1.

**Fig. 2:**
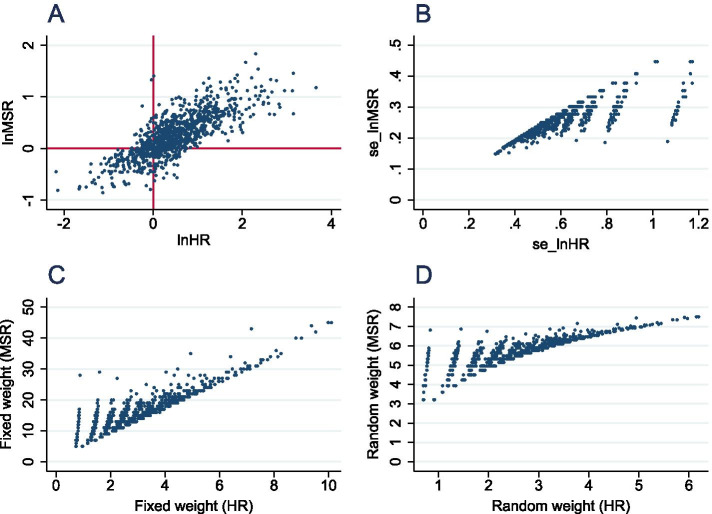
Head-to-head comparison of *HR*- and *MSR*-based summary statistics. Scatter plots comparing efficacy estimates (**A**), standard errors (**B**), fixed-effects weightings (**C**) and random-effects weighting as calculated in DerSimonian and Laird meta-analysis (**D**)

The *stcox* function successfully generated *HR* estimates for all 1000 experiments in the first simulation, with a median of 1.52 (*IQR* 1.02–2.62), although a small percentage returned extreme *HR*s favouring either treatment (11/1000, 1.1%) or control (1/1000, 0.1%) with correspondingly large standard error (see Supplementary Material 3 for examples). These were not excluded as their weighting would be diminished at meta-analysis. The median *MSR* for the dataset was 1.25 (*IQR* 0.97–1.70)—there were no extreme outliers, range 0.423–6.27. On log-transforming each statistic prior to application to meta-analysis, there was a modest correlation between ln*HR* and ln*MSR* (*r* = 0.37, Fig. 2A). The same direction of treatment effect was suggested by both summary statistics in 840/1000 (84%) of instances; opposite treatment effects were suggested in the remaining 160 (16%). Typically, in these instances, the efficacy estimates were modest for both measures and differing polarities could be accounted for by non-significant treatment effect or crossing Kaplan-Meier curves (see Supplementary Material 4).

The measure of error used for ln*HR* was the standard error generated from the *stcox* estimation. As *MSR* measurement does not inherently produce a measure of error, this is estimated using the number of animals in the experiment as a surrogate. The total number of individuals in the experiment is used in place of inverse variance as a meta-analysis weighting factor, so SE for ln*MSR* is thus estimated using 1/√*n*. There was a linear correlation between se_ln*HR* and se_ln*MSR* although standard errors were larger and of greater range for *HR* data (Fig. 2B). Correspondingly, there was a correlation in fixed-effects weighting with weighting values being larger for *MSR* data than for *HR*, and absolute values much higher for *MSR* data than *HR* (Fig. 2C). Consequently, the *τ*^2^ estimate for the MSR-based meta-analysis was 0.111, compared with 0.0626 for the *HR* approach—meaning that random-effects weighting was more consistent in the *MSR* meta-analysis (Fig. 2D).

Meta-analysis of *HR* data suggested a significant treatment effect with pooled *HR* of 1.50 (*95% CI* 1.44–1.56; *t* = 19.3, *p* < 0.001). Similarly, there was pooled *MSR* of 1.29 (*95% CI* 1.26–1.32; *t* = 19.0, *p* < 0.001). The *I*^2^ values were high for *MSR* data (63.7%) and low for *HR* data (23.5%). On univariate meta-regression, a significant predictive effect of 6 variables was identified using *HR* (Var1, Var2, Var3, Bin1, Bin2) and of 5 using *MSR* (Var1, Var2, Var3, Bin1, Bin2, Cont1). Similarly, multivariate meta-regression of these 1000 studies revealed the predictive value of 5 variables for both *HR* and *MSR* (Var1, Var2, Var3, Bin1, Bin2).

To summarise, we have found strong correlations between the *HR* and *MSR* summary statistics as well as their performance in a single large meta-analysis.

### Meta-analysis and meta-regression power assessment

The simulation was repeated for 100,000 experiments containing 1.6m individuals in order to allow for an assessment of the sensitivity of each approach by repeated meta-analyses. This was done using the same parameters as the first simulation, except for the number of individuals and experiments. The median study group size was 8 (*IQR* 7–10). Death occurred for 98.7% of individuals during the experiment and the overall median survival was 8.39 days.

Cox regression again suggested a significant influence of treatment, with *HR* 1.46 ± 0.0237, *Z* = 234 and *p* < 0.001. Median survival was 9.42 days in the treatment group and 7.55 in the control, giving a *MSR* of 1.25. Similarly, Cox regression suggested a comparable influence of each predictive variable on survival outcome to those in the first dataset and no influence of the control variables (see Supplementary Material 5).

The iteration failed to converge in 34 instances (0.034%) of *HR* estimation and so these experiments were excluded from the remainder of the simulation. These experiments mostly had exceptionally small sample sizes (see Supplementary Material 6 for examples). Whenever a meta-analysis had a study excluded, it was still treated as if it were its original size (that is, of size 20 experiments instead of 19 or 100 instead of 99). There were no instances where a single meta-analysis had 2 experiments excluded.

**Fig. 3:**
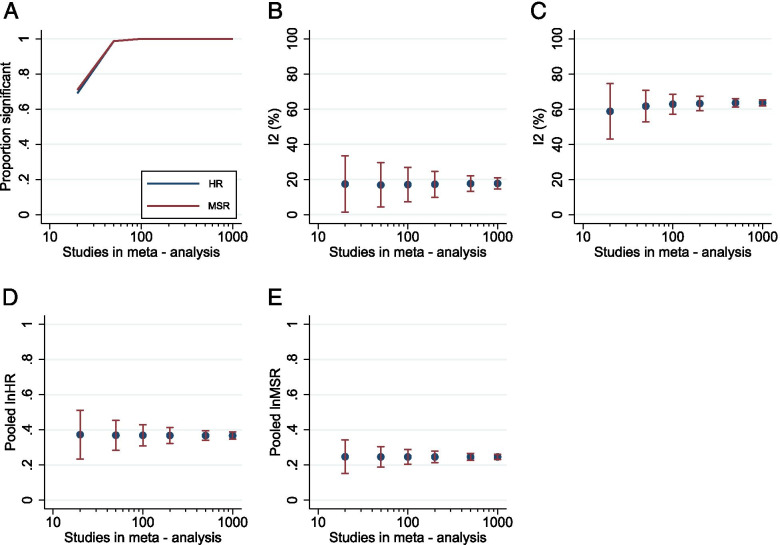
Comparison of performance of *HR*- and *MSR*-based summary statistics at meta-analysis. Plots showing sensitivity of meta-analyses of size 20–1000 studies to detect treatment effect (**A**; *α* = 0.05); *I*^2^ across the range of meta-analysis sizes for *HR* (**B**) and *MSR* (**C**); and global efficacy estimates for *HR* (**D**) and *MSR* (**E**). Plots represent the mean ± *SD*

*HR*- and *MSR*-based summary statistics performed similarly at random-effects meta-analysis. Their ability to detect a treatment effect was comparable, with sensitivity around 70% of meta-analysis of 20 experiments for each summary statistic and close to 100% for those including 50 or more experiments (Fig. 3A). *I*^2^ values were consistently low for *HR*-based meta-analyses, with few values returned over 25% (Fig. 3B), in keeping with the more conservative SE estimations discussed above. Conversely, *I*^2^ was consistently between 60 and 65% throughout the range of meta-analysis sizes for *MSR* data (Fig. 3C). The *I*^2^ was higher for *MSR*-based approaches in every instance than HR. There was a fairly consistent global efficacy estimation across both datasets, with variance of estimates slightly lower for the *MSR* meta-analyses (Fig. 3D, E).

**Fig. 4:**
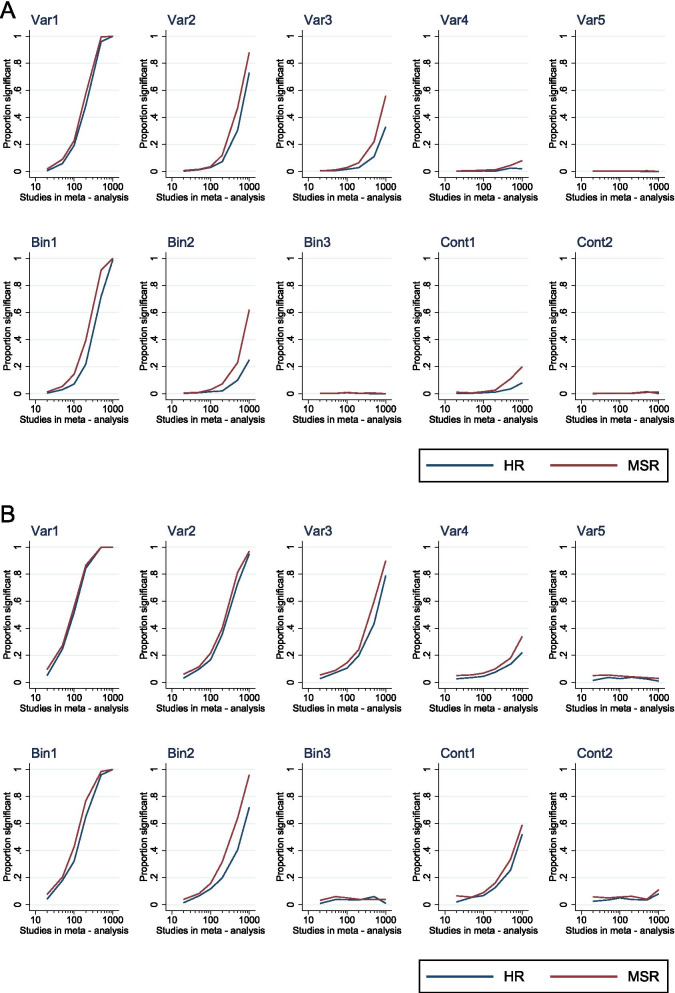
Grouped plots showing sensitivity of meta-regression to detect the influence of each variable at univariate (**A**; *α* = 0.005) and multivariate (**B**; *α* = 0.05) stages. Each plot shows the proportion of significant associations (*α* as specified above) versus the meta-analysis size. Blue plots denote *HR*-based meta-regression, red denotes *MSR*-based meta-regression; Var1-5 represent the categorical variables, Bin1-2 represent the binary variables and Cont1-2 represent the continuous variables

We compared the ability of meta-regression to detect the predictive value of covariates on treatment outcome for both *MSR*- and *HR*-based meta-analysis, at both univariate and multivariate stages. At every stage, alpha was set to 0.005 for univariate meta-regressions to account for multiple testing, and 0.05 for multivariate meta-regressions. For univariate meta-regression, sensitivity was overall relatively low. The power to detect even the stronger associations (for example, with Var1 and Bin1) was below 50% in meta-analyses of 100 studies or less for each dataset. However, sensitivity increased to over 80% for strong associations at 200 studies and moderate associations (e.g. Var2, Var3, Bin2) at 1000 studies. There was no major advantage of one summary statistic over the other in terms of sensitivity, although *MSR*-based meta-analysis slightly outperformed *HR*-based meta-analysis in every case. The type I error rate was maintained around 0.05 for each of the control variables throughout the range of meta-analysis sizes (Var5, Bin3, Cont2; Fig. 4A).

Finally, we undertook the same assessment but using multivariate meta-regression. Again, sensitivity was relatively low but not dissimilar to that found at univariate meta-regression. Power was limited to below 50% (*α* 0.05) for even strong associations when meta-analysis size was 100 or less, but a power of 80% or more was seen for strong and moderate associations for larger meta-analyses in the same manner as the univariate meta-regression (Fig. 4B). With *α* set as above, multivariate meta-regression appeared to confer a sensitivity advantage over univariate meta-regression (see Supplementary Material 7). Indeed, if *α* is increased to 0.05 for both strategies, there were no appreciable differences in sensitivity between to two techniques for *MSR* data (data not shown).

This section shows that, on repeated meta-analysis and meta-regression, the power for *MSR* to detect associations is equivalent if not superior to *HR* for small studies.

### Publication bias inclusion

We recreated the large dataset (*n* = 1.6m individuals, 100,000 experiments) but introduced a file drawer effect to simulate publication biases of varying strengths by randomly discarding 0%, 25%, 50%, 75% and 100% of experiments for which there was no apparent treatment effect on log-rank testing. Of the 100,000 experiments, only 17,076 (17.1%) returned a significant log-rank test statistic. Thus, the sample size reduced for each dataset as the influence of the file drawer effect increased. That being said, meta-analyses were only included in the analysis if their size was at least 90% of that intended (18 for group size 20, 900 for group size 1000, etc.).

**Fig. 5:**
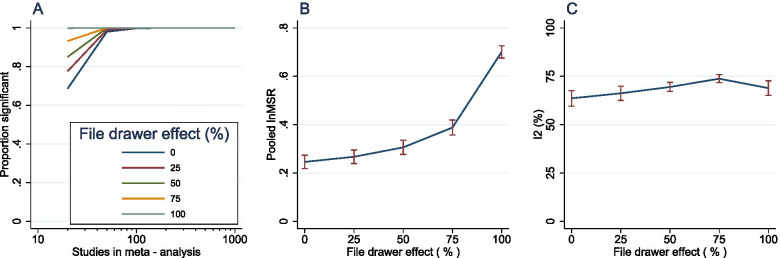
Comparison of *MSR*-based meta-analyses in the context of varying degrees of file drawer effect. Plots showing the sensitivity of meta-analyses of size 20–1000 studies to detect treatment effect (**A**; *α* = 0.05); global efficacy estimates (**B**) and *I*^2^ (**C**) across the range of meta-analysis sizes. Plots represent the mean ± *SD*

Unsurprisingly, the sensitivity of meta-analysis to detect treatment effect increased with increasing file drawer effect (Fig. 5A) despite a dramatic reduction in sample size. Because global efficacy estimates appeared not to vary greatly between different meta-analysis sizes, we compared global efficacy estimates and *I*^2^ for meta-analyses of size 1000 only as these returned estimates with the greatest precision. There was a dramatic increase in the perceived global efficacy estimates as publication bias influence increased, with median survival ratios of 2.01 (ln*MSR* = 0.702) observed in studies in which all non-significant experiments were discarded and 1.47 (ln*MSR* = 0.387) with a file drawer effect of 75% (Fig. 5B). There was no clear association between the file drawer effect and between-study heterogeneity (Fig. 5C).

**Fig. 6:**
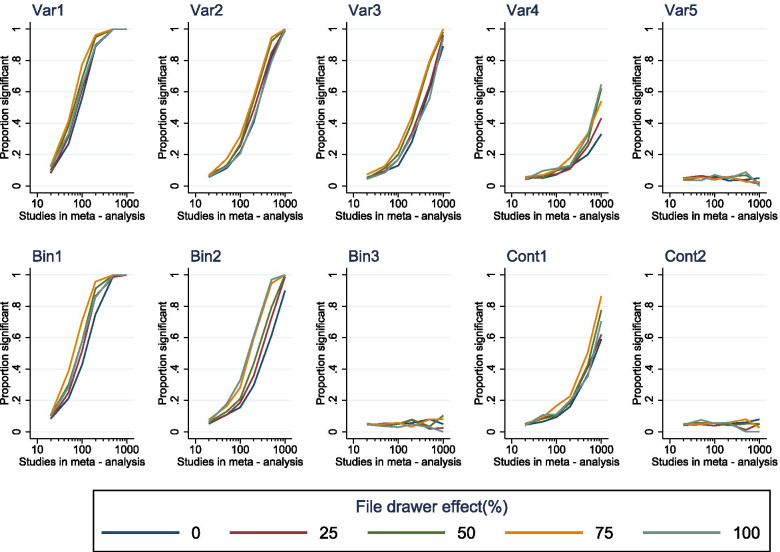
Grouped plots showing the sensitivity of *MSR* meta-regression to detect the influence of each variable at multivariate stage (*α* = 0.05) across a range of file drawer effects. Each plot shows the proportion of significant associations versus meta-analysis size. Var1-5 represent the categorical variables, Bin1-2 represent the binary variables and Cont1-2 represent the continuous variables

We compared the performance of multivariate meta-regression of *MSR* data compromised by varying file drawer effects. While there was no major correlation between file drawer influence and meta-regression sensitivity, there was a trend for more biased datasets to suggest slightly higher power for detection of associations of any strength, without a clear increase in the type I error rate (Fig. 6).

This section gives an estimation of the true influence of publication bias, which cannot be accurately measured from real data (as the problem is missing/irretrievable data).

## Discussion

In this study, we have explored the use of the median survival ratio as a convenient alternative to hazard ratio using a preclinical meta-analysis simulation. We believe that, for meta-analyses including large numbers of small, imprecise studies, we have validated *MSR* use as a summary statistic. We have undertaken multiple meta-analyses and provided a power assessment of meta-regression at both univariate and multivariate phases and believe that this is informative for future preclinical survival meta-analyses.

### Survival simulation

Our study used a series of survival data, all generated with the same parameters—that is, with the same influence of treatment and other variables. We chose the treatment effect deliberately to give a *MSR* in the range of 1.25–1.3, as this is what we have encountered in previous meta-analyses of animal glioblastoma studies [25–27]. A useful exercise might be to appraise the comparative performance of *HR* vs *MSR* across a range of treatment effect sizes. However, we directed our attention toward the meta-regression phase of analysis as this is where the focus lies in preclinical meta-analysis. We included 10 variables into the survival model, with 7 having predictive effects on treatment outcome. We achieved this by including the variables a separate hazards in the *survsim* command, so as to ensure an interaction between each variable and the treatment variable, as simply including 10 variables as covariates along with the treatment variable does not generate this interaction [29]. We confirmed this interaction by demonstrating significant proportional hazards on Cox regression of raw survival data, as well as by the demonstration of associations in meta-regressions. We hoped to input a range of realistic associations while keeping the survival data tangible, although we acknowledge that the outcome of power analysis is directly affected by the magnitude of associations generated at this stage. Our ‘strong’ associations, for example with the first binary variable, were associated with a 12% difference in *MSR* which probably underestimates those seen in real datasets.

A significant limitation in this survival simulation is the lack of collinearity. We have raised concerns of collinearity in prior survival meta-analyses (for example certain immune conditions being required for different tumour paradigms or different delivery methods for different drugs) which can return false results via confounding effects. We had hoped to document the type I error rate by including variables with no influence on survival or treatment outcome, but were unable to effectively introduce collinearity into this simulation. We have recommended multivariate approaches for preclinical glioblastoma meta-analyses on this basis and our power studies are favourable in this regard. Secondly, our simulation does not account for ‘unknown unknowns’, or factors of influence which cannot be measured. This is a significant issue in preclinical meta-analysis and only acts to create additional heterogeneity that cannot be explained. Because this simulation is not affected by this effect, our estimations for power in meta-analysis and meta-regression may be less for real data. It is impossible to quantify the effect of unknown unknowns on real meta-analyses, so attempts to introduce this effect in survival simulation with appropriate magnitude will be precarious.

We chose to limit the study to a length where the majority of individuals had died, as this is often the case in experimental studies (for control data at least). Shortening the timeframe to, say, 30 days where around 25% of individuals survive might be more realistic as continuing experiments beyond the point of equipoise is generally considered unethical. A truncation like this is unlikely to affect *MSR* (as median survival would still be observed in the majority of experiments), but *HR* assessment could change: a shortened timeframe would give fewer timepoints for comparison, fewer events and more censored data and differences in long-term survivorship could be overlooked [16, 20]. *HR* estimates would therefore be less precise, compromising meta-analysis and meta-regression sensitivity—thus further favouring the use of *MSR*.

### Validity of summary statistics

The *HR*-based strategy appeared to be computationally more challenging than for *MSR*, and as an iterative technique, there were a subset (around 1%) of studies for which estimations were either outliers or an estimate could not be given. Both of these issues are presumably a consequence of the small sample sizes and could generate problems for meta-analysis, skewing results. That being said, this problem was not limited to studies of particularly small sample size. Extreme efficacy estimates—which appear to be a product of the *HR* estimation process rather than the grouping of individuals into studies as the variance was similarly high and corresponding *MSR*s within expected range—may skew meta-analysis unduly with the introduction of type I errors during meta-regression. However, in this simulation, the variance of these *HR*s is correspondingly large and these studies are weighted lightly, especially given the small *τ*^2^ values associated with *HR* meta-analyses. Experiments for which iteration fails to converge on a *HR* estimate must be omitted from meta-analysis—conferring an arguably avoidable risk of bias. One advantage of hazard-based survival analysis is the inclusion of censored data, but this is rarely used in experimental studies because of the controlled nature of their circumstances.

Median survival ratios were computationally more straightforward to calculate and are much simpler to estimate from Kaplan-Meier charts than *HR*s. They have appeared to produce more consistent efficacy estimates and we did not observe extreme or missing results. The fundamental limitation of this metric is the lack of a measure of spread—the substitute measure of precision (via study size) has likely led to underestimations of efficacy estimate variance, meaning that heterogeneity is overestimated. This is reflected in the very high *I*^2^ values seen in *MSR*-based meta-analyses. Random-effects weighting has therefore tended more toward a straight average as *τ*^*2*^ is high, meaning that meta-analysis moves more toward an unweighted average. This is advantageous in quelling doubt around the validity of precision estimation, but does put meta-regression at risk of inflated influence from individual outlying, imprecise studies.

Outliers excluded, *HR* and *MSR* gave analogous results in the majority of instances. There were 16% of studies for which the polarity of *HR* and *MSR* were opposite, which is comparable with the observations made by Michiels et al. [23]. However, in this simulation, efficacy estimates were small in every case; for these 160 experiments, none was associated with a significant treatment effect of Cox regression or log-rank testing (*α* = 0.05). As such, the issue of differing efficacy estimate polarities does not appear to be a major issue in the meta-analysis of very small studies.

### Performance at meta-analysis

Despite more conservative estimates of efficacy and precision in the *HR* dataset, and clear differences in heterogeneity estimation, we were surprised with the similarity in meta-analysis and meta-regression power between the two summary statistics. As expected, the power to detect a treatment effect was high throughout the range of meta-analysis sizes, except for those combining only 20 experiments.

Meta-regression was limited in sensitivity, in keeping with prior concerns [10, 11]. We did not expect to observe comparable performance between *HR* and *MSR* at both univariate and multivariate stages, especially given the large discrepancy in heterogeneity estimation. *MSR* had a slight advantage across all variables which may reflect the greater observed heterogeneity when using this technique. Regardless, this did not appear to come at the cost of an increase in type I error rates. Multivariate meta-regression in preclinical meta-analyses is often limited as studies are excluded due to collinearity, resulting in a compromise in sensitivity that is not accounted in this simulation.

We have demonstrated, for both summary statistics, that with this level of variable influence, meta-regression is of little use for meta-analyses smaller than 100 experiments; indeed, for confidence in the detection of moderate and strong associations, a number closer to 500 is likely to be needed. In the preclinical literature, single treatments are seldomly reported in 500 instances. As such, to reliably use meta-regression as a tool to investigate study design and quality features for time-to-event data, we believe that the pooling of multiple treatments is required to ensure the appropriate level of power. This would require a multivariate approach, because different treatments of course have differing efficacies and a univariate approach may be unreliable in the context of these unaccounted confounding effects. Network meta-analysis has proven useful when comparing multiple treatments and its application to survival data may be useful in this regard [32].

### Publication bias assessment

Using this set of parameters, we observed a 17% power of studies to detect treatment effect, which is similar to an estimate made previously from real data [25]. Introducing a file drawer effect appears to have, as expected, generate greater perceived efficacy estimates and resulted in a higher proportion of small meta-analyses detecting treatment effect.

Meta-analyses of the most biased datasets returned perceived efficacy much higher than the original dataset, in keeping with publication bias phenomena observed in preclinical and clinical meta-analyses. While techniques have been described to accommodate for this or even to estimate its influence, they are known to be insensitive. Indeed, applying Trim and Fill analysis to a single meta-analysis of size 1000 experiments in the context of a 100% file drawer effect suggested a reduction of ln*MSR* from 0.690 to 0.502 only—far short of the mean treatment effect of 0.246 observed in a meta-analysis of unbiased data.

We thought including more experiments favouring positive treatment effect might lead to a reduced heterogeneity estimate as studies suggested efficacy more consistently, although this was not the case and *I*^2^ remained fairly consistent across the range of file drawer effects. Our strategy was designed to exclude non-significant study results but did not filter for those favouring a positive treatment effect; that is, our biased datasets did include some studies that suggested a significant negative treatment effect. However, this was the case for only 988 experiments (0.9% of the entire dataset; 5.8% of the significant log-rank tests)—so we believe that this would not fundamentally change the findings of this analysis.

Meta-regression of biased datasets appeared to show a slight sensitivity advantage over those without publication bias in some circumstances—although this was not a sustained, clear correlation. This association may be explained by the fact that subtle influences of predictive variables are easier to detect when there is less noise in the range of treatment effects. However, a relatively constant *I*^2^ would refute this. A second explanation could be that this apparent increased sensitivity could represent a type I error. There appeared to be no clear trend for an increased type I error rate in control variables, although in some instances significant associations were seen as frequently as 10%. If meta-regression sensitivity is inappropriately increased in biased meta-analyses, the data from this study suggests that the problem is marginal and would not radically affect the findings.

## Conclusions

In this simulation, we believe we have demonstrated that, for meta-analyses of small experimental studies, median survival ratio is at least equivalent in sensitivity and reliability to hazard ratio-based techniques, while being simpler to calculate and with more consistent summary statistic calculation process and output. As such, its use in preclinical meta-analyses is advisable and practical. Although concerns around the use of study size as a measure of precision remain valid, we have not observed any detrimental consequences—conversely, it appears to result in an increase in meta-regression sensitivity. Similarly, median survival ratios can suggest the opposite direction of treatment effect to hazard ratio in larger trials, but this does not appear to be a major issue in the meta-analysis of small, imprecise studies. We have provided an estimation of meta-regression sensitivity in a single set of conditions which suggests that large meta-analyses are likely required in order to appropriately manage the risk of type II error. Finally, we have shown that multivariate approaches are superior in terms of sensitivity and that their performance is not fundamentally compromised by severe publication bias.

## Supplementary Information

**Additional file 1: Supplementary Material 1.** Code used for all simulations and analyses.

**Additional file 2: Supplementary Material 2.** Histogram of experiment group sizes. Individuals in experiments were divided equally into treatment and control groups.

**Additional file 3: Supplementary Material 3.** Example Kaplan-Meier curves for experiments returning extreme HR values.

**Additional file 4: Supplementary Material 4.** Example Kaplan-Meier curves for experiments returning HR and MSR in opposite polarities.

**Additional file 5: Supplementary Material 5.** Output from Cox regression of individual survival data in second simulation (n=1.6m).

**Additional file 6: Supplementary Material 6.** Example Kaplan-Meier curves for experiments where HR iteration failed to converge.

**Additional file 7: Supplementary Material 7.** Grouped plots showing sensitivity of MSR meta-regression to detect influence of each variable at univariate (red; α=0.005) and multivariate (blue; α=0.05) stages. Each plot shows the proportion of significant associations (α as specified above) versus meta-analysis size.

## Data Availability

All data generated or analysed during this study are included in this published article [and its supplementary information files].
