# Supplementary material for: Using median survival in meta-analysis of experimental time-to-event data
Source: Syst Rev. 2021 Nov 2;10:292. doi: 10.1186/s13643-021-01824-0 (PMC8561932; doi:10.1186/s13643-021-01824-0)
Supplement: Supplementary file 1 — Additional file 1: Supplementary Material 1. Code used for all simulations and analyses. [file 13643_2021_1824_MOESM1_ESM.docx]

* code for 1st meta-analysis simulation

*This includes 1000 studies of mean n=8 per grp, 16 per experiment

*set directory for data*

cd "C:\data\Main simulation"

*generate study characteristics database*

clear all

set seed 1889

set obs 1000

seq study

gen var1 = ceil(5*uniform())

gen var2 = ceil(5*uniform())

gen var3 = ceil(5*uniform())

gen var4 = ceil(5*uniform())

gen var5 = ceil(5*uniform())

gen bin1 = rbinomial(1,0.5)

gen bin2 = rbinomial(1,0.5)

gen bin3 = rbinomial(1,0.5)

gen cont1 = rnormal(6,2)

gen cont2 = rnormal(6,2)

save studyfeats, replace

*gen individual survival data*

clear

set seed 1889

set obs 16000

generate study = 1+round(999*rbeta(1,0.8))

sort study

by study: gen N=_N

seq trt, f(0) t(1)

*import variables from study features dataset*

merge m:1 study using studyfeats

drop _merge

*survival time simulation*

survsim stime state event, hazard1(distribution(weibull) lambda (0.1) gamma (1.5) covariates (trt 8)) hazard2(distribution(weibull) lambda (0.1) gamma (2) covariates (var1 2.5)) hazard3(distribution(weibull) lambda (0.1) gamma (2) covariates (var2 2.2)) hazard4(distribution(weibull) lambda (0.1) gamma (2) covariates (var3 2.1)) hazard5(distribution(weibull) lambda (0.1) gamma (2) covariates (var4 1.9)) hazard6 (distribution(weibull) lambda (0.1) gamma (2) covariates (var5 0)) hazard7(distribution(weibull) lambda (0.1) gamma (2) covariates (bin1 10.5)) hazard8(distribution(weibull) lambda (0.1) gamma (2) covariates (bin2 9.5)) hazard9 (distribution(weibull) lambda (0.1) gamma (2) covariates (bin3 0)) hazard10(distribution(weibull) lambda (0.1) gamma (2) covariates (cont1 1.1)) hazard11(distribution(weibull) lambda (0.1) gamma (2) covariates (cont2 0)) maxtime(0.05)

stset stime1, scale(0.001)

save individualsurvdata, replace

*calculate MSRs from individual data file and save to tempfile*

clear all

use individualsurvdata

su study, meanonly

postfile msrtemp lnmsr selnmsr N study using "msrtemp.dta", replace

_dots 0, title(Loop running) reps(1000)

forvalues i = 1/`r(max)' {

qui: summarize _t if study == `i'

local n = r(N)

qui: summarize _t if study == `i' & trt == 1, detail

qui: return list

local medc = r(p50)

qui: summarize _t if study == `i' & trt == 0, detail

qui: return list

local medrx = r(p50)

local lnmsr = log(`medrx'/`medc')

local se1 = sqrt(`n')

local se = 1/(`se1')

post msrtemp (`lnmsr') (`se') (`n') (`i')

_dots `i' 0

}

postclose msrtemp

*calculate HRs and save to tempfile*

statsby lnhr = _b[trt] selnhr = _se[trt], by(study) saving(hrtemp, replace): stcox trt

* merge hrtemp and msrtemp onto variable file*

use studyfeats

merge 1:1 study using "msrtemp.dta"

drop _merge

merge 1:1 study using "hrtemp.dta"

drop _merge

save studyfeats, replace

erase msrtemp.dta

erase hrtemp.dta

*drop if HR not recorded as causes error in metaregression process; implies that HR not calculated anyway*

drop if selnhr == 0

*display 1/SE, tau, random effects weighting for each study

local PE "lnmsr lnhr"

foreach X in `PE' {

qui: capture metareg `X', wsse(se`X')

local tau = e(tau2)

gen `X'tau = `tau'

gen `X'_rwt = 1/((se`X'^2) + `X'tau)

}

gen lnhr_precision = 1/selnhr

gen lnmsr_precision = N

gen lnhr_fwt = 1/(selnhr^2)

gen lnmsr_fwt = N

save studyfeats, replace

*cox regression of individual data, saving file with proportional HRs*

clear all

use individualsurvdata

postfile hrcox trt var1 var2 var3 var4 var5 bin1 bin2 bin3 cont1 cont2 using "cox_hrs.dta", replace

stcox trt var1 var2 var3 var4 var5 bin1 bin2 bin3 cont1 cont2

mat r = r(table)

local trt = r[1,1]

local v1 = r[1,2]

local v2 = r[1,3]

local v3 = r[1,4]

local v4 = r[1,5]

local v5 = r[1,6]

local b1 = r[1,7]

local b2 = r[1,8]

local b3 = r[1,9]

local c1 = r[1,10]

local c2 = r[1,11]

post hrcox (`trt') (`v1') (`v2') (`v3') (`v4') (`v5') (`b1') (`b2') (`b3') (`c1') (`c2')

postclose hrcox

* looping code for power calculation/2^nd^ meta-analysis

*This includes 100000 studies of average n=8 per grp, 16 per study

*Split into meta-analyses of 20, 50, 100, 200, 500, 1000 studies with comparison of the power of median survival ratio and hazard ratio to detect variable influence on metaregression outcome

*

*set directory for data*

cd "C:\data\Power simulation"

*generate study characteristics database*

clear all

set seed 1889

set obs 100000

seq study

gen var1 = ceil(5*uniform())

gen var2 = ceil(5*uniform())

gen var3 = ceil(5*uniform())

gen var4 = ceil(5*uniform())

gen var5 = ceil(5*uniform())

gen bin1 = rbinomial(1,0.5)

gen bin2 = rbinomial(1,0.5)

gen bin3 = rbinomial(1,0.5)

gen cont1 = rnormal(6,2)

gen cont2 = rnormal(6,2)

save studyfeats, replace

*gen individual survival data*

clear

set seed 1889

set obs 1600000

generate study = 1+round(99999*rbeta(1,0.8))

sort study

by study: gen N=_N

seq trt, f(0) t(1)

*import variables from study features dataset*

merge m:1 study using studyfeats

drop _merge

*survival time simulation*

survsim stime state event, hazard1(distribution(weibull) lambda (0.1) gamma (1.5) covariates (trt 8)) hazard2(distribution(weibull) lambda (0.1) gamma (2) covariates (var1 2.5)) hazard3(distribution(weibull) lambda (0.1) gamma (2) covariates (var2 2.2)) hazard4(distribution(weibull) lambda (0.1) gamma (2) covariates (var3 2.1)) hazard5(distribution(weibull) lambda (0.1) gamma (2) covariates (var4 1.9)) hazard6 (distribution(weibull) lambda (0.1) gamma (2) covariates (var5 0)) hazard7(distribution(weibull) lambda (0.1) gamma (2) covariates (bin1 10.5)) hazard8(distribution(weibull) lambda (0.1) gamma (2) covariates (bin2 9.5)) hazard9 (distribution(weibull) lambda (0.1) gamma (2) covariates (bin3 0)) hazard10(distribution(weibull) lambda (0.1) gamma (2) covariates (cont1 1.1)) hazard11(distribution(weibull) lambda (0.1) gamma (2) covariates (cont2 0)) maxtime(0.05)

stset stime1, scale(0.001)

save individualsurvdata, replace

*calculate MSRs from individual data file and save to tempfile*

clear all

use individualsurvdata

su study, meanonly

postfile msrtemp lnmsr selnmsr study using "msrtemp.dta", replace

_dots 0, title(Loop running) reps(100000)

forvalues i = 1/`r(max)' {

qui: summarize _t if study == `i'

local n = r(N)

qui: summarize _t if study == `i' & trt == 1, detail

qui: return list

local medc = r(p50)

qui: summarize _t if study == `i' & trt == 0, detail

qui: return list

local medrx = r(p50)

local lnmsr = log(`medrx'/`medc')

local se1 = sqrt(`n')

local se = 1/(`se1')

post msrtemp (`lnmsr') (`se') (`i')

_dots `i' 0

}

postclose msrtemp

*calculate HRs and save to tempfile*

statsby lnhr = _b[trt] selnhr = _se[trt], by(study) saving(hrtemp, replace): stcox trt

* merge hrtemp and msrtemp onto variable file*

use studyfeats

merge 1:1 study using "msrtemp.dta"

drop _merge

merge 1:1 study using "hrtemp.dta"

drop _merge

*drop if HR not recorded as causes error in metaregression process; implies that HR not calculated anyway*

drop If selnhr == 0

*assign randomly into meta-analysis groups of size 20-1000*

gen temp = runiform()

sort temp

seq grp20, b(20)

drop temp

gen temp = runiform()

sort temp

seq grp50, b(50)

drop temp

gen temp = runiform()

sort temp

seq grp100, b(100)

drop temp

gen temp = runiform()

sort temp

seq grp200, b(200)

drop temp

gen temp = runiform()

sort temp

seq grp500, b(500)

drop temp

gen temp = runiform()

sort temp

seq grp1000, b(1000)

drop temp

sort study

save studyfeats, replace

erase msrtemp.dta

erase hrtemp.dta

*calculate global efficacy estimates, I2 and significance for meta-analyses of size 20-1000 studies*

*limit of 1000 meta-regressions for each group size*

clear all

use studyfeats

tempfile working

save working, replace

local PE "lnhr lnmsr"

postfile buffer str20 Statistic str20 Groupsize Subgroup N p I2 PE using "MApower.dta", replace

foreach Y in `PE' {

local G "grp20 grp50 grp100 grp200 grp500 grp1000"

foreach X in `G' {

use working

su `X', meanonly

local c = cond(`r(max)' > 1000, 1000, `r(max)')

forvalues i = 1/`c' {

use working

display "working on_MA_""`Y'" "_""`X'" "_" `i'

qui: keep if `X' == `i'

capture metareg `Y', wsse(se`Y') difficult

local subgrp = `i'

mat results = r(table)

local N = e(N)

local I2 = e(I2)

local grpsize = "`X'"

local p = results[4,1]

local PE = results[1,1]

post buffer ("`Y'") ("`grpsize'") (`subgrp') (`N') (`p') (`I2') (`PE')

clear

}

}

}

postclose buffer

*calculate summary file for MA power*

clear all

use MApower

postfile buffer str20 Statistic Groupsize MApower I2 I2sd PE PEsd using "MApowersummary.dta", replace

tab Statistic, gen(PE)

tab Groupsize, gen(gs)

gen MAgrp = cond(gs1==1,100,cond(gs2==1,1000,cond(gs3==1,20,cond(gs4==1,200,cond(gs5==1,50,500)))))

local grp "gs3 gs5 gs1 gs4 gs6 gs2"

tempfile working

save working, replace

forvalues i = 1/2 {

clear

use working

keep if PE`i' == 1

tempfile working2

save working2, replace

foreach X in `grp' {

clear

use working2

keep if `X' == 1

qui: count

local N = r(N)

local grpsize = MAgrp

qui: count if p < 0.05

qui: return list

local stat = Statistic

local sig = r(N)

local p = `sig'/`N'

qui: summarize I2

local i2 = r(mean)

local i2sd = r(sd)

qui: summarize PE

local pe = r(mean)

local pesd = r(sd)

post buffer ("`stat'") (`grpsize') (`p') (`i2') (`i2sd') (`pe') (`pesd')

}

}

postclose buffer

*create summary graphs for MA output*

clear all

use MApowersummary

tab Statistic, gen(s)

twoway (line MApower Groupsize if s1==1, lwidth(0.4)) (line MApower Groupsize if s2==1, lwidth(0.4)), nodraw graphregion(fcolor(white) lcolor(white) lstyle(none)) title(Meta-analysis sensitivity, pos(11) ring (-0.5)) xsc(log range(10 1000)) xtitle (Studies in meta-analysis) xlabel (10 100 1000) xmtick(20 30 40 50 60 70 80 90 200 300 400 500 600 700 800 900) ysc(r(0 1)) ytitle("Proportion significant") ylabel(0(0.2)1) legend(pos(5) ring(0) size(vsmall) cols(1) lab(1 "HR") lab(2 "MSR")) name(MApow, replace)

clear

use MApower

tab Statistic, gen(s)

tab Groupsize, gen(gs)

gen MAgrp = cond(gs1==1,100,cond(gs2==1,1000,cond(gs3==1,20,cond(gs4==1,200,cond(gs5==1,50,500)))))

local grp "gs3 gs5 gs1 gs4 gs6 gs2"

gen i2 = I2*100

tempfile working

save working, replace

keep if s1 == 1

collapse (mean) mean=i2 (sd) sd=i2, by(MAgrp)

gen hi = mean+sd

gen lo = mean-sd

*calculate p values for multivariate metaregression of size of 20-1000 studies*

*limit of 1000 meta-regressions for each group size*

clear all

use studyfeats

tempfile working

save working, replace

local PE "lnhr lnmsr"

postfile buffer str20 Statistic str20 Groupsize Subgroup N Var1p Var2p Var3p Var4p Var5p Bin1p Bin2p Bin3p Cont1p Cont2p using "mmrpower.dta", replace

foreach Y in `PE' {

local G "grp20 grp50 grp100 grp200 grp500 grp1000"

foreach X in `G' {

use working

su `X', meanonly

local c = cond(`r(max)' > 1000, 1000, `r(max)')

forvalues i = 1/`c' {

use working

display "working on_mmr_""`Y'" "_""`X'" "_" `i'

qui: keep if `X' == `i'

capture metareg `Y' var1 var2 var3 var4 var5 bin1 bin2 bin3 cont1 cont2, wsse(se`Y') difficult

local subgrp = `i'

mat results = r(table)

local N = e(N)

local grpsize = "`X'"

local Var1p = results[4,1]

local Var2p = results[4,2]

local Var3p = results[4,3]

local Var4p = results[4,4]

local Var5p = results[4,5]

local Bin1p = results[4,6]

local Bin2p = results[4,7]

local Bin3p = results[4,8]

local Cont1p = results[4,9]

local Cont2p = results[4,10]

post buffer ("`Y'") ("`grpsize'") (`subgrp') (`N') (`Var1p') (`Var2p') (`Var3p') (`Var4p') (`Var5p') (`Bin1p') (`Bin2p') (`Bin3p') (`Cont1p') (`Cont2p')

clear

}

}

}

postclose buffer

*calculate p values for univariate metaregression of size of 20-1000 studies*

*limit of 1000 meta-regressions for each group size*

clear all

use studyfeats

tempfile working

save working, replace

local PE "lnhr lnmsr"

postfile buffer str20 Statistic str20 Groupsize Subgroup N Var1p Var2p Var3p Var4p Var5p Bin1p Bin2p Bin3p Cont1p Cont2p using "umrpower.dta", replace

foreach Y in `PE' {

use working

local G "grp20 grp50 grp100 grp200 grp500 grp1000"

foreach X in `G' {

use working

su `X', meanonly

local c = cond(`r(max)' > 1000, 1000, `r(max)')

forvalues i = 1/`c' {

use working

display "working on_umr_""`Y'" "_""`X'" "_" `i'

qui: keep if `X' == `i'

local var "var1 var2 var3 var4 var5 bin1 bin2 bin3 cont1 cont2"

foreach var in `var' {

capture metareg `Y' `var', wsse(se`Y') difficult

local subgrp = `i'

mat results = r(table)

local N = e(N)

local grpsize = "`X'"

local `var'p = results[4,1]

}

post buffer ("`Y'") ("`grpsize'") (`subgrp') (`N') (`var1p') (`var2p') (`var3p') (`var4p') (`var5p') (`bin1p') (`bin2p') (`bin3p') (`cont1p') (`cont2p')

clear

}

}

}

postclose buffer

*create summary power file for mmr data*

clear all

use mmrpower

postfile buffer str20 Statistic Groupsize Var1 Var2 Var3 Var4 Var5 Bin1 Bin2 Bin3 Cont1 Cont2 using "mmrpowersummary.dta", replace

tab Statistic, gen(PE)

local var "Var1p Var2p Var3p Var4p Var5p Bin1p Bin2p Bin3p Cont1p Cont2p"

tab Groupsize, gen(gs)

gen MAgrp = cond(gs1==1,100,cond(gs2==1,1000,cond(gs3==1,20,cond(gs4==1,200,cond(gs5==1,50,500)))))

local grp "gs3 gs5 gs1 gs4 gs6 gs2"

tempfile working

save working, replace

forvalues i = 1/2 {

clear

use working

keep if PE`i' == 1

tempfile working2

save working2, replace

foreach X in `grp' {

clear

use working2

keep if `X' == 1

qui: count

local N = r(N)

local grpsize = MAgrp

local stat = Statistic

foreach Y in `var' {

qui: count if `Y' < 0.05

qui: return list

local sig = r(N)

local `Y'ow = `sig'/`N'

}

post buffer ("`stat'") (`grpsize') (`Var1pow') (`Var2pow') (`Var3pow') (`Var4pow') (`Var5pow') (`Bin1pow') (`Bin2pow') (`Bin3pow') (`Cont1pow') (`Cont2pow')

}

}

postclose buffer

*create summary power file for umr data*

clear all

use umrpower

postfile buffer str20 Statistic Groupsize Var1 Var2 Var3 Var4 Var5 Bin1 Bin2 Bin3 Cont1 Cont2 using "umrpowersummary.dta", replace

tab Statistic, gen(PE)

local var "Var1p Var2p Var3p Var4p Var5p Bin1p Bin2p Bin3p Cont1p Cont2p"

tab Groupsize, gen(gs)

gen MAgrp = cond(gs1==1,100,cond(gs2==1,1000,cond(gs3==1,20,cond(gs4==1,200,cond(gs5==1,50,500)))))

local grp "gs3 gs5 gs1 gs4 gs6 gs2"

tempfile working

save working, replace

forvalues i = 1/2 {

clear

use working

keep if PE`i' == 1

tempfile working2

save working2, replace

foreach X in `grp' {

clear

use working2

keep if `X' == 1

qui: count

local N = r(N)

local grpsize = MAgrp

local stat = Statistic

foreach Y in `var' {

qui: count if `Y' < 0.05

qui: return list

local sig = r(N)

local `Y'ow = `sig'/`N'

}

post buffer ("`stat'") (`grpsize') (`Var1pow') (`Var2pow') (`Var3pow') (`Var4pow') (`Var5pow') (`Bin1pow') (`Bin2pow') (`Bin3pow') (`Cont1pow') (`Cont2pow')

}

}

postclose buffer

erase working.dta

erase working2.dta

*combine datasets

clear all

use umrpowersummary

gen analysis = 2

save umrpowersummary, replace

use mmrpowersummary

gen analysis =1

save mmrpowersummary.dta, replace

append using umrpowersummary

save "combpowersummary.dta", replace

erase mmrpowersummary.dta

erase umrpowersummary.dta

* code for publication bias effect

*This includes 100000 studies of mean n=8 per grp, 16 per experiment

*many similarities between this and second dataset, can just copy individualsurvdata and studyfeats over to save on computation time if required

*set directory for data*

cd "C:\data\pubbias"

*generate study characteristics database*

clear all

set seed 1889

set obs 100000

seq study

gen var1 = ceil(5*uniform())

gen var2 = ceil(5*uniform())

gen var3 = ceil(5*uniform())

gen var4 = ceil(5*uniform())

gen var5 = ceil(5*uniform())

gen bin1 = rbinomial(1,0.5)

gen bin2 = rbinomial(1,0.5)

gen bin3 = rbinomial(1,0.5)

gen cont1 = rnormal(6,2)

gen cont2 = rnormal(6,2)

save studyfeats, replace

*gen individual survival data*

clear

set seed 1889

set obs 1600000

generate study = 1+round(99999*rbeta(1,0.8))

sort study

by study: gen N=_N

seq trt, f(0) t(1)

*import variables from study features dataset*

merge m:1 study using studyfeats

drop _merge

*survival time simulation*

survsim stime state event, hazard1(distribution(weibull) lambda (0.1) gamma (1.5) covariates (trt 8)) hazard2(distribution(weibull) lambda (0.1) gamma (2) covariates (var1 2.5)) hazard3(distribution(weibull) lambda (0.1) gamma (2) covariates (var2 2.2)) hazard4(distribution(weibull) lambda (0.1) gamma (2) covariates (var3 2.1)) hazard5(distribution(weibull) lambda (0.1) gamma (2) covariates (var4 1.9)) hazard6 (distribution(weibull) lambda (0.1) gamma (2) covariates (var5 0)) hazard7(distribution(weibull) lambda (0.1) gamma (2) covariates (bin1 10.5)) hazard8(distribution(weibull) lambda (0.1) gamma (2) covariates (bin2 9.5)) hazard9 (distribution(weibull) lambda (0.1) gamma (2) covariates (bin3 0)) hazard10(distribution(weibull) lambda (0.1) gamma (2) covariates (cont1 1.1)) hazard11(distribution(weibull) lambda (0.1) gamma (2) covariates (cont2 0)) maxtime(0.05)

stset stime1, scale(0.001)

save individualsurvdata, replace

*calculate MSRs from individual data file and save to tempfile*

clear all

use individualsurvdata

su study, meanonly

postfile msrtemp lnmsr selnmsr N study using "msrtemp.dta", replace

_dots 0, title(Loop running) reps(1000)

forvalues i = 1/`r(max)' {

qui: summarize _t if study == `i'

local n = r(N)

qui: summarize _t if study == `i' & trt == 1, detail

qui: return list

local medc = r(p50)

qui: summarize _t if study == `i' & trt == 0, detail

qui: return list

local medrx = r(p50)

local lnmsr = log(`medrx'/`medc')

local se1 = sqrt(`n')

local se = 1/(`se1')

post msrtemp (`lnmsr') (`se') (`n') (`i')

_dots `i' 0

}

postclose msrtemp

*do logrank test and give p values*

clear all

use individualsurvdata

su study, meanonly

save working, replace

postfile hrtemp LRchi p study using "hrtemp.dta", replace

_dots 0, title(Loop running) reps(100000)

forvalues i = 1/`r(max)' {

use working, clear

qui: keep if study == `i'

qui: sts test trt

local chi = r(chi2)

local p = chi2tail(1,`chi')

local study = `i'

post hrtemp (`chi') (`p') (`i')

_dots `i' 0

}

postclose hrtemp

* merge hrtemp and msrtemp onto variable file*

use studyfeats, clear

merge 1:1 study using "msrtemp.dta"

drop _merge

merge 1:1 study using "hrtemp.dta"

drop _merge

save studyfeats, replace

erase msrtemp.dta

erase hrtemp.dta

*delete sig results accordingly 0-100. PB100 means all studies p>0.05 deleted, PB75 is 75% nonsig studies deleted at random, etc*

set seed 1889

use studyfeats, clear

keep if p<0.05

save sig, replace

use studyfeats, clear

keep if p>=0.05

gen r = uniform()

qui: count

local grp = r(N)

local g2 = ceil(`grp'/4)

seq r2, b(`g2')

save nsig, replace

forvalues i=0/4 {

use nsig

drop if r2 <= `i'

local n = (`i'*25)

append using sig

drop r r2

save PB`n', replace

}

*for each dataset, assign randomly into meta-analysis groups of size 20-1000*

local PE "PB0 PB25 PB50 PB75 PB100"

foreach D in `PE' {

use `D'

gen temp = runiform()

sort temp

seq grp20, b(20)

drop temp

gen temp = runiform()

sort temp

seq grp50, b(50)

drop temp

gen temp = runiform()

sort temp

seq grp100, b(100)

drop temp

gen temp = runiform()

sort temp

seq grp200, b(200)

drop temp

gen temp = runiform()

sort temp

seq grp500, b(500)

drop temp

gen temp = runiform()

sort temp

seq grp1000, b(1000)

drop temp

sort study

save `D', replace

}

erase sig.dta

erase nsig.dta

*calculate global efficacy estimates, I2 and significance for meta-analyses of size 20-1000 studies*

*limit of 1000 meta-regressions for each group size*

clear all

local PE "PB0 PB25 PB50 PB75 PB100"

postfile buffer str20 PBeffect str20 Groupsize Subgroup N p I2 PE using "MApower.dta", replace

foreach Y in `PE' {

use `Y'

tempfile working

save working, replace

local G "grp20 grp50 grp100 grp200 grp500 grp1000"

foreach X in `G' {

use working

su `X', meanonly

local c = cond(`r(max)' > 1000, 1000, `r(max)')

forvalues i = 1/`c' {

use working

display "working on_MA_""`Y'" "_""`X'" "_" `i'

qui: keep if `X' == `i'

capture metareg lnmsr, wsse(selnmsr) difficult

local subgrp = `i'

mat results = r(table)

local N = e(N)

local I2 = e(I2)

local grpsize = "`X'"

local p = results[4,1]

local PE = results[1,1]

post buffer ("`Y'") ("`grpsize'") (`subgrp') (`N') (`p') (`I2') (`PE')

clear

}

}

}

postclose buffer

*calculate summary file for MA power*

clear all

use MApower

postfile buffer PBeffect Groupsize MApower I2 I2sd PE PEsd using "MApowersummary.dta", replace

tab PBeffect, gen(pb)

gen a = cond(pb1==1,0,cond(pb2==1,100,cond(pb3==1,25,cond(pb4==1,50,75))))

local Z "pb1 pb2 pb3 pb4 pb5"

tab Groupsize, gen(gs)

gen MAgrp = cond(gs1==1,100,cond(gs2==1,1000,cond(gs3==1,20,cond(gs4==1,200,cond(gs5==1,50,500)))))

keep if N == MAgrp

local grp "gs3 gs5 gs1 gs4 gs6 gs2"

tempfile working

save working, replace

su `pb', meanonly

foreach C in `Z'{

clear

use working

keep if `C' == 1

tempfile working2

save working2, replace

foreach X in `grp' {

clear

use working2

keep if `X' == 1

qui: count

local N = r(N)

local grpsize = MAgrp

local A = a

qui: count if p < 0.05

qui: return list

local sig = r(N)

local p = `sig'/`N'

qui: summarize I2

local i2 = r(mean)

local i2sd = r(sd)

qui: summarize PE

local pe = r(mean)

local pesd = r(sd)

post buffer (`A') (`grpsize') (`p') (`i2') (`i2sd') (`pe') (`pesd')

}

}

postclose buffer

*create summary graphs for MA output*

clear all

use MApowersummary

tab PBeffect, gen(s)

twoway (line MApower Groupsize if s1==1, lwidth(0.4)) (line MApower Groupsize if s2==1, lwidth(0.4)) (line MApower Groupsize if s3==1, lwidth(0.4)) (line MApower Groupsize if s4==1, lwidth(0.4)) (line MApower Groupsize if s5==1, lwidth(0.4)), graphregion(fcolor(white) lcolor(white) lstyle(none)) title(Meta-analysis sensitivity, pos(11) ring (-0.5)) xsc(log range(10 1000)) xtitle (Studies in meta-analysis) xlabel (10 100 1000) xmtick(20 30 40 50 60 70 80 90 200 300 400 500 600 700 800 900) ysc(r(0 1)) ytitle("Proportion significant") ylabel(0(0.2)1) legend(pos(5) ring(0) title(File drawer effect (%)) size(med) cols(1) lab(1 "0") lab(2 "25") lab(3 "50") lab(4 "75") lab(5 "100")) name(MApow, replace)

clear

**graph of PE vs MA size for different PB effects**

use MApower, clear

tab PBeffect, gen(pb)

tab Groupsize, gen(gs)

gen MAgrp = cond(gs1==1,100,cond(gs2==1,1000,cond(gs3==1,20,cond(gs4==1,200,cond(gs5==1,50,500)))))

keep if MAgrp == 1000

gen a = cond(pb1==1,0,cond(pb2==1,100,cond(pb3==1,25,cond(pb4==1,50,75))))

collapse (mean) mean=PE (sd) sd=PE, by(MAgrp a)

gen hi = mean+1.96*sd

gen lo = mean-1.96*sd

twoway (line mean a, lwidth(0.4)) (rcap lo hi a), legend(off) graphregion(fcolor(white) lcolor(white) lstyle(none)) title(PE in publication-biased MSR meta-analyses, pos(11) ring (-0.5)) xtitle (File drawer effect (%)) xlabel(0(25)100) ytitle("Pooled lnMSR") ylabel(0(0.2)0.8) name(PE_PBeffect, replace)

clear

**graph of I2 vs MA size for different PB effects**

use MApower, clear

tab PBeffect, gen(pb)

tab Groupsize, gen(gs)

gen MAgrp = cond(gs1==1,100,cond(gs2==1,1000,cond(gs3==1,20,cond(gs4==1,200,cond(gs5==1,50,500)))))

keep if MAgrp == 1000

gen a = cond(pb1==1,0,cond(pb2==1,100,cond(pb3==1,25,cond(pb4==1,50,75))))

collapse (mean) mean=I2 (sd) sd=I2, by(MAgrp a)

gen hi = 100*(mean+1.96*sd)

gen lo = 100*(mean-1.96*sd)

gen I2 = 100*mean

twoway (line I2 a, lwidth(0.4)) (rcap lo hi a), legend(off) graphregion(fcolor(white) lcolor(white) lstyle(none)) title(I2 in publication-biased MSR meta-analyses, pos(11) ring (-0.5)) xtitle (File drawer effect (%)) xlabel(0(25)100) ytitle("I2 (%)") ylabel(0(25)100) name(I2_PBeffect, replace)

clear

graph combine MApow PE_PBeffect I2_PBeffect, rows(1) cols(3) graphregion(fcolor(white)) ysize(3in) xsize(8in) saving(publicationbiaseffect, replace)

*calculate p values for multivariate metaregression of size of 20-1000 studies*

*limit of 1000 meta-regressions for each group size*

clear all

local PE "PB0 PB25 PB50 PB75 PB100"

postfile buffer str20 PBeffect str20 Groupsize Subgroup N Var1p Var2p Var3p Var4p Var5p Bin1p Bin2p Bin3p Cont1p Cont2p using "mmrpower.dta", replace

foreach Y in `PE' {

use `Y'

tempfile working

save working, replace

local G "grp20 grp50 grp100 grp200 grp500 grp1000"

foreach X in `G' {

use working

su `X', meanonly

local c = cond(`r(max)' > 1000, 1000, `r(max)')

forvalues i = 1/`c' {

use working

display "working on_mmr_""`Y'" "_""`X'" "_" `i'

qui: keep if `X' == `i'

capture metareg lnmsr var1 var2 var3 var4 var5 bin1 bin2 bin3 cont1 cont2, wsse(selnmsr) difficult

local subgrp = `i'

mat results = r(table)

local N = e(N)

local grpsize = "`X'"

local Var1p = results[4,1]

local Var2p = results[4,2]

local Var3p = results[4,3]

local Var4p = results[4,4]

local Var5p = results[4,5]

local Bin1p = results[4,6]

local Bin2p = results[4,7]

local Bin3p = results[4,8]

local Cont1p = results[4,9]

local Cont2p = results[4,10]

post buffer ("`Y'") ("`grpsize'") (`subgrp') (`N') (`Var1p') (`Var2p') (`Var3p') (`Var4p') (`Var5p') (`Bin1p') (`Bin2p') (`Bin3p') (`Cont1p') (`Cont2p')

clear

}

}

}

postclose buffer

*create summary power file for mmr data*

clear all

use mmrpower

postfile buffer PBeffect Groupsize Var1 Var2 Var3 Var4 Var5 Bin1 Bin2 Bin3 Cont1 Cont2 using "mmrpowersummary.dta", replace

tab PBeffect, gen(pb)

gen a = cond(pb1==1,0,cond(pb2==1,100,cond(pb3==1,25,cond(pb4==1,50,75))))

local Z "pb1 pb2 pb3 pb4 pb5"

local var "Var1p Var2p Var3p Var4p Var5p Bin1p Bin2p Bin3p Cont1p Cont2p"

tab Groupsize, gen(gs)

gen MAgrp = cond(gs1==1,100,cond(gs2==1,1000,cond(gs3==1,20,cond(gs4==1,200,cond(gs5==1,50,500)))))

keep if N >= 0.9*MAgrp

local grp "gs3 gs5 gs1 gs4 gs6 gs2"

tempfile working

save working, replace

su `pb', meanonly

foreach C in `Z'{

clear

use working

keep if `C' == 1

tempfile working2

save working2, replace

foreach X in `grp' {

clear

use working2

keep if `X' == 1

local A = a

qui: count

local N = r(N)

local grpsize = MAgrp

foreach Y in `var' {

qui: count if `Y' < 0.05

qui: return list

local sig = r(N)

local `Y'ow = `sig'/`N'

}

post buffer (`A') (`grpsize') (`Var1pow') (`Var2pow') (`Var3pow') (`Var4pow') (`Var5pow') (`Bin1pow') (`Bin2pow') (`Bin3pow') (`Cont1pow') (`Cont2pow')

}

}

postclose buffer

*create graphs from mmrpowersummary file*

clear all

use mmrpowersummary

tab PBeffect, gen(s)

local var " Var1 Var2 Var3 Var4 Var5 Bin1 Bin2 Bin3 Cont1 Cont2"

foreach X in `var' {

qui: twoway (line `X' Groupsize if s1==1, lwidth(0.4)) (line `X' Groupsize if s2==1, lwidth(0.4)) (line `X' Groupsize if s3==1, lwidth(0.4)) (line `X' Groupsize if s4==1, lwidth(0.4)) (line `X' Groupsize if s5==1, lwidth(0.4)), nodraw aspectratio(1.75) graphregion(fcolor(white) lcolor(white) lstyle(none)) title(`X', pos(11) ring (-0.5)) xsc(log range(10 1000)) xtitle (Studies in meta-analysis) xlabel (10 100 1000) xmtick(20 30 40 50 60 70 80 90 200 300 400 500 600 700 800 900) ysc(r(0 1)) ytitle("Proportion significant") ylabel(0(0.2)1) legend(title (File drawer effect) size(small) rows(1) lab(1 "0") lab(2 "25") lab(3 "50") lab(4 "75") lab(5 "100")) name(`X', replace)

}

grc1leg `var', rows(2) cols(5) graphregion(fcolor(white)) ysize(6in) xsize(12in) iscale(*1.075) pos(5) ring(2) saving(mmrpowersummary, replace)
