# Supplementary figures and images for: Using median survival in meta-analysis of experimental time-to-event data
Source: Syst Rev. 2021 Nov 2;10:292. doi: 10.1186/s13643-021-01824-0 (PMC8561932; doi:10.1186/s13643-021-01824-0)

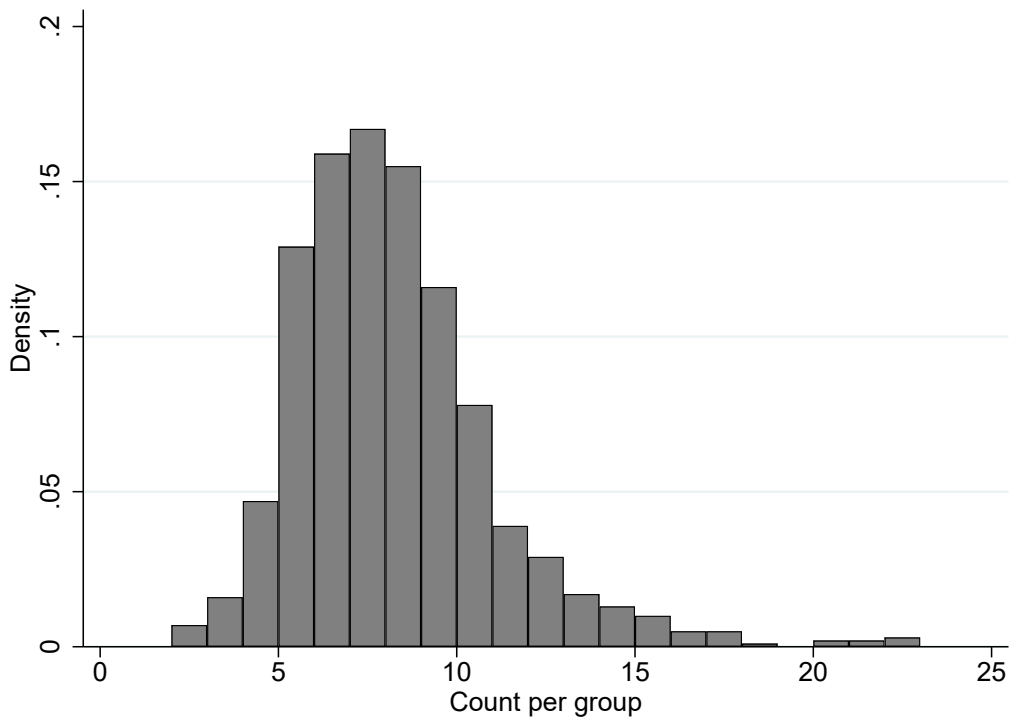

Supplement: Supplementary file 2 — Additional file 2: Supplementary Material 2. Histogram of experiment group sizes. Individuals in experiments were divided equally into treatment and control groups. [file 13643_2021_1824_MOESM2_ESM.pdf]

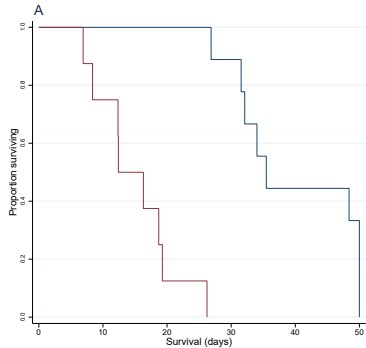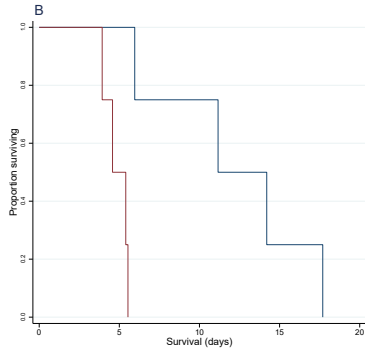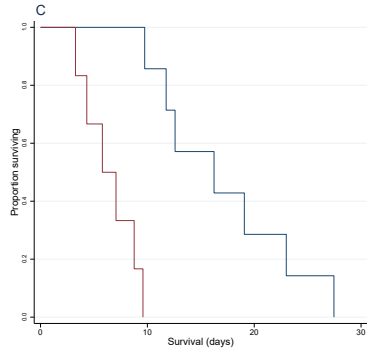

Supplement: Supplementary file 3 — Additional file 3: Supplementary Material 3. Example Kaplan-Meier curves for experiments returning extreme HR values. [file 13643_2021_1824_MOESM3_ESM.pdf]

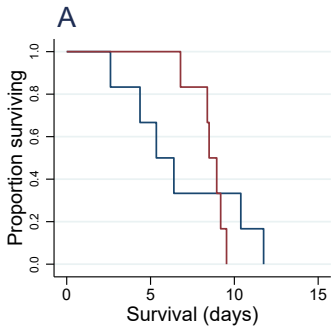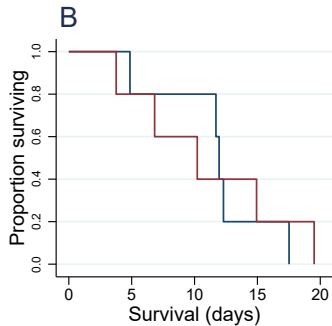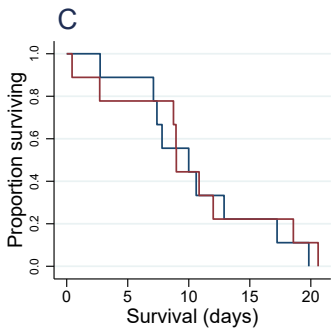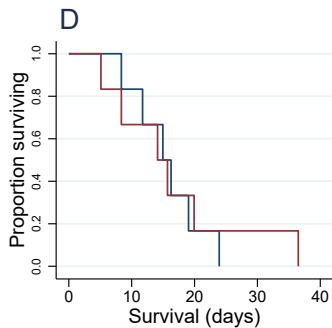

Supplement: Supplementary file 4 — Additional file 4: Supplementary Material 4. Example Kaplan-Meier curves for experiments returning HR and MSR in opposite polarities. [file 13643_2021_1824_MOESM4_ESM.pdf]

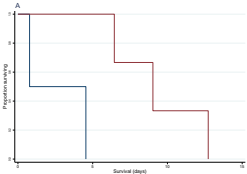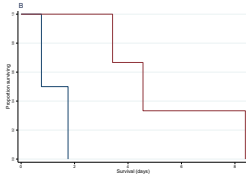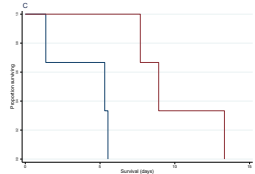

Supplement: Supplementary file 6 — Additional file 6: Supplementary Material 6. Example Kaplan-Meier curves for experiments where HR iteration failed to converge. [file 13643_2021_1824_MOESM6_ESM.pdf]

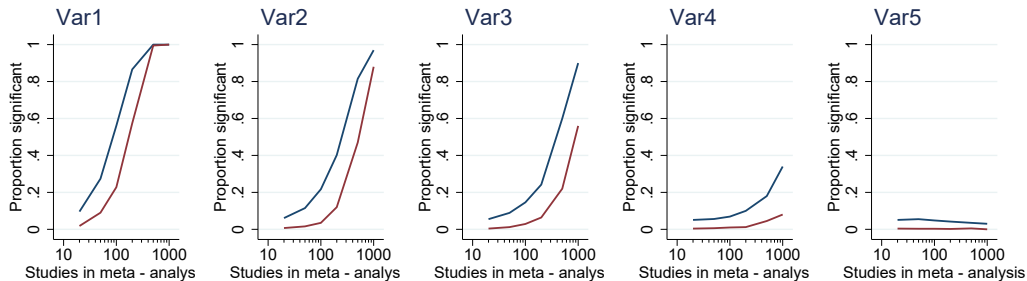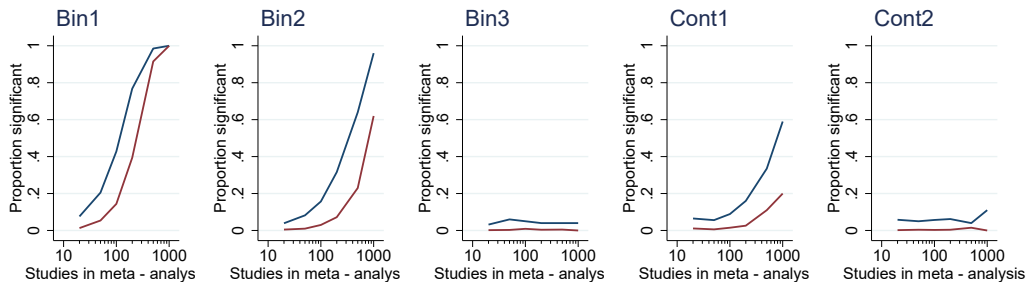

— MMR — UMR

Supplement: Supplementary file 7 — Additional file 7: Supplementary Material 7. Grouped plots showing sensitivity of MSR meta-regression to detect influence of each variable at univariate (red; α=0.005) and multivariate (blue; α=0.05) stages. Each plot shows the proportion of significant associations (α as specified above) versus meta-analysis size. [file 13643_2021_1824_MOESM7_ESM.pdf]
