# Supplementary material for: Using median survival in meta-analysis of experimental time-to-event data
Source: Syst Rev. 2021 Nov 2;10:292. doi: 10.1186/s13643-021-01824-0 (PMC8561932; doi:10.1186/s13643-021-01824-0)
Supplement: Supplementary file 5 — Additional file 5: Supplementary Material 5. Output from Cox regression of individual survival data in second simulation (n=1.6m). [file 13643_2021_1824_MOESM5_ESM.docx]

------------------------------------------------------------------------------

_t | Haz. Ratio Std. Err. z P>|z| [95% Conf. Interval]

-------------+----------------------------------------------------------------

trt | 1.462274 .0023666 234.79 0.000 1.457643 1.46692

var1 | 1.370769 .0009214 469.19 0.000 1.368964 1.372576

var2 | 1.142018 .0006876 220.57 0.000 1.140671 1.143367

var3 | 1.09997 .0006465 162.12 0.000 1.098704 1.101238

var4 | 1.045074 .0005958 77.33 0.000 1.043907 1.046242

var5 | 1.000743 .0005605 1.33 0.185 .9996447 1.001842

bin1 | 1.693877 .0028234 316.19 0.000 1.688352 1.699419

bin2 | 1.261419 .002029 144.38 0.000 1.257448 1.265402

bin3 | .9989298 .0015796 -0.68 0.498 .9958387 1.00203

cont1 | 1.052889 .0004481 121.10 0.000 1.052012 1.053768

cont2 | 1.000035 .0003976 0.09 0.929 .9992565 1.000815

------------------------------------------------------------------------------
